# Research Trends, Hotspots and Future Perspectives of Geometric Morphometrics in Entomology: A Scientometric Review

**DOI:** 10.3390/insects17030325

**Published:** 2026-03-17

**Authors:** Yusha Tan, Zihui Zhao, Xiaojuan Yuan, Yuanqi Zhao, Di Su, Yuehua Song

**Affiliations:** 1 School of Karst Science, Guizhou Normal University, Guiyang 550025, China; tanyusha2024@163.com (Y.T.); zhaozihui2000@163.com (Z.Z.); yxj00816@163.com (X.Y.); zyq19991208@163.com (Y.Z.); sudibao@163.com (D.S.); 2 State Engineering Technology Institute for Karst Desertification Control, Guiyang 550025, China

**Keywords:** geometric morphometrics, entomology, scientometrics, knowledge mapping

## Abstract

Geometric morphometrics is an important component of quantitative research on insect morphology, widely applied in taxonomy, intraspecific variation, and phylogenetic studies. However, systematic research in this field remains limited, with few comprehensive summaries of research trends, hotspots, and core theories. This study, based on scientometric methods, analyzed 1321 publications indexed in the Web of Science database up to 31 December 2025, and presents a meta-scientific review from a macro perspective, revealing the research trends, hotspots, and future directions in the field. The results show that: (1) annual publications exhibit overall growth, while research methods evolved from single landmark analysis to multimodal and interdisciplinary approaches; (2) scientists from Brazil, the USA, and France are major contributors, with studies spanning morphology, taxonomy, and ecology; (3) taxonomic studies centered on wing shape analysis constitutes a major research hotspot, closely related to phylogeny, allometry, and sexual dimorphism; (4) highly co-cited studies provide the main theoretical and methodological foundations for the field. Future research, building on existing hotspots, will further integrate geometric morphometrics with genomics, ecological functional data, three-dimensional geometric morphometrics, and artificial intelligence-assisted approaches to advance integrative taxonomy within interdisciplinary and data-driven frameworks.

## Simple Summary

Geometric morphometrics is increasingly used in insect morphology research and has become a powerful tool in taxonomy, intraspecific variation, and phylogenetic studies. However, the overall development of this field has received little attention. In this study, we analyzed 1321 papers to summarize the application of geometric morphometrics in insect research over the past three decades. The results reveal rapid growth in publication output, with major contributions from Brazil, the USA, and France. Wing shape-based taxonomy represents a major research hotspot and is closely connected with phylogeny, allometry, and sexual dimorphism. This study summarizes the current research landscape and future directions, offering valuable references for researchers regarding methodological selection, research design, and interdisciplinary integration, and may further promote advances in insect biodiversity research and conservation practice.

## 1. Introduction

Morphology is a fundamental basis for species identification and taxonomic research and serves as an important biological indicator of environmental variation, playing a pivotal role in biological research [1]. In the early stages of taxonomy, researchers primarily relied on qualitative descriptions of discontinuous morphological differences; however, such descriptions were highly subjective and often failed to reach broad consensus, particularly when dealing with complex structures [2]. In the mid-20th century, the rise of cladistics established a relatively objective framework for morphological research, while the widespread application of biostatistical methods promoted the development of morphometrics [3]. By integrating multivariate statistical techniques, morphometrics reveals fundamental information such as organismal size and shape, marking the transition from qualitative morphological description to quantitative morphological analysis [4]. However, traditional morphometrics focuses primarily on statistical measurement data and is insufficient for analyzing shape variation alone, as linear measurements often fail to capture the geometric relationships of morphological structures [5]. Geometric morphometrics, proposed by Rohlf and others, emerged during the 1980s and 1990s and has since developed rapidly, being widely applied in taxonomy, ecology, and other fields [6]. This method is based on Cartesian coordinate data, using geometric morphometrics to capture organismal form variation, quantify morphological traits, and enable multivariate statistical analyses, thereby enabling these traits to be incorporated into traditional taxonomy and phylogenetic analyses [7,8]. The main approaches in geometric morphometrics include the landmark method and the outline method. The former employs homologous points of insects to establish a series of coordinate landmarks, while the latter is used when sufficient landmarks cannot be defined, by selecting points along the contour of insect structures in a specified orientation [9,10]. Compared with traditional morphometrics, this method is less affected by non-shape information and reduces bias introduced by subjective factors [11].

In the field of entomology, geometric morphometrics is widely applied to taxonomy, intraspecific variation analysis, phylogenetic reconstruction, and fossil morphological studies [12,13]. For many insects with rigid exoskeletons that undergo limited morphological change after reaching adulthood, geometric morphometric approaches based on multiple quantitative traits provide an effective means of characterizing such variation [14]. Compared with the outline method, the landmark-based method offers several advantages, including efficient dimensionality reduction, robustness to morphological variation, high cross-study comparability, and relatively low computational cost, which contribute to its broader application [15,16,17,18]. When the number of landmarks on biological structures is insufficient for analysis, or when the structures exhibit smooth contours or curved features, semi-landmarks are often combined with landmarks [19,20,21,22]. With advances in computational techniques, an increasing range of analytical methods has been introduced in geometric morphometrics, such as Partial Least Squares, Generalized Procrustes Superimposition, and analyses of morphological integration and modularity. These approaches have been applied to study geographic variation in insect morphology, insect phylogeny, and taxonomy [23,24,25,26].

Despite the rapid development of geometric morphometrics and the availability of numerous methodological descriptions and application-oriented studies, systematic reviews in this field remain limited. However, a systematic literature review in this research field remains insufficient, and there is a lack of comprehensive analysis of the characteristics of published literature in geometric morphometrics. Therefore, this study employs scientometric methods to conduct a quantitative and visual analysis of 1321 publications on insect geometric morphometrics included in the Web of Science database as of 31 December 2025. We further summarize the main research methods, viewpoints, and applications in this field and discuss the broader implications of geometric morphometrics for entomological research.

## 2. Materials and Methods

### 2.1. Data Acquisition and Selection Process

The data used in this study were retrieved from the Web of Science (WoS) Core Collection. To ensure both accuracy and comprehensiveness, the advanced search function of WoS was employed, with the specific search criteria defined as follows: TS = (“Geometr* Morpho*”) AND TS = (Insect OR Entomology OR Insecta OR Siphonaptera OR Coleoptera OR Lepidoptera OR Orthoptera OR Diptera OR Hemiptera OR Homoptera OR Isoptera OR Hymenoptera OR Dermaptera OR Neuroptera OR Plecoptera OR Thysanoptera OR Protura OR Sinentomata OR Archaeognatha OR Crustacea OR Phyllopoda OR Branchiopoda OR Malacostraca OR Maxillopoda OR Arachnida OR Araneae OR Gnaphosidae OR Acari OR Trematoda OR Hirudinea OR Rhynchobdellida OR Arhynchobdellida OR Oligochaeta OR Polychaeta OR Phyllodocida OR Nereidiformia OR Sabellida OR Sarcodina OR Acantharia OR Spumellaria OR Granuloreticulosa OR Foraminifera OR Dorylaimida OR Rhabditida OR Hydroidomedusa OR Siphonophorae OR Cystonectae OR Physonectae OR Calycophorae OR Polycystinea OR Nassellaria OR Phaeodarea OR Phaeodaria OR Sagittoidea OR Aphragmophora OR Oligohymenophorea OR Peritrichida OR Phascolosomatidea OR Sipunculidea OR Golfingiiformes OR “Sipunculus nudus Linnaeus” OR Echiuroidea OR Aplousobranchia). This search strategy was designed to maximize the retrieval of studies related to geometric morphometrics in insects while minimizing the risk of omitting relevant publications. The search was limited to records indexed before 31 December 2025. During the careful screening and verification of the search results, we focused on sorting and analyzing the literature related to the application of geometric morphometrics in entomology. Publications from disciplines not directly relevant to the scope of this study were excluded, including anthropology, genetics, medicine, oceanography, psychology, applied physics, and other fields. Ultimately, 1321 empirical research articles were selected as the dataset for this study.

### 2.2. Research Methods

This study employs scientometric methods to visualize and analyze data retrieved from WoS. Scientometrics is an interdisciplinary field that applies mathematical, statistical, and data-analytical techniques to quantitatively investigate scientific and technological activities [27]. Beyond citation analysis, it also plays a significant role in evaluating research outputs [28]. Compared with traditional bibliometrics, scientometrics offers greater depth and breadth: it enables the visualization of authors, countries, publication volume, and citations, while also revealing their interrelationships through knowledge maps. These maps systematically present the intrinsic connections and dynamic changes within the literature, allowing for a clear and objective identification of research trends in insect geometric morphometrics and providing valuable references for future studies.

**Figure 1:**
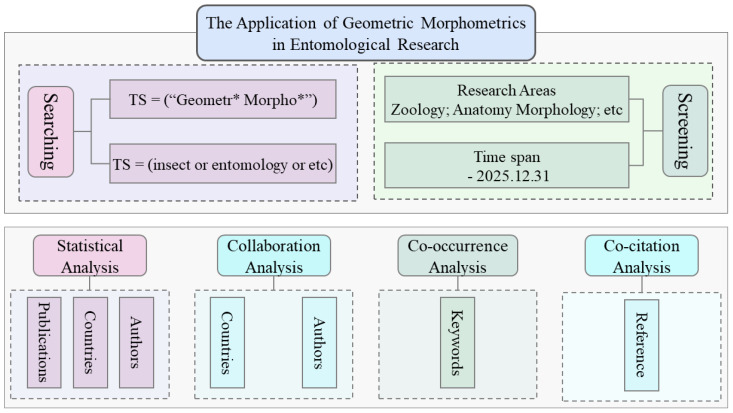
Scientometric analysis workflow.

Currently, scientometric analyses mainly rely on software such as CiteSpace, VOSviewer, and HistCite [29,30,31,32,33]. These tools support co-occurrence, collaboration and co-citation analyses across multiple knowledge units and allow the construction of knowledge maps for authors, publication volume, keywords, and more [34,35]. In this study, records retrieved from WoS were exported in plain-text format with “full records and cited references” and processed using CiteSpace (version 6.3.R1), VOSviewer (version 1.6.20), and Scimago Graphica (version 1.0.50). We constructed knowledge maps of the literature related to geometric morphometrics and insects published up to 31 December 2025. The analyses include statistical analysis (annual publications, countries, authors), collaboration analysis (authors, countries), co-occurrence analysis (keywords), and co-citation analysis (references). The overall workflow is shown in Figure 1.

## 3. Results

### 3.1. Statistical Analysis

**Figure 2:**
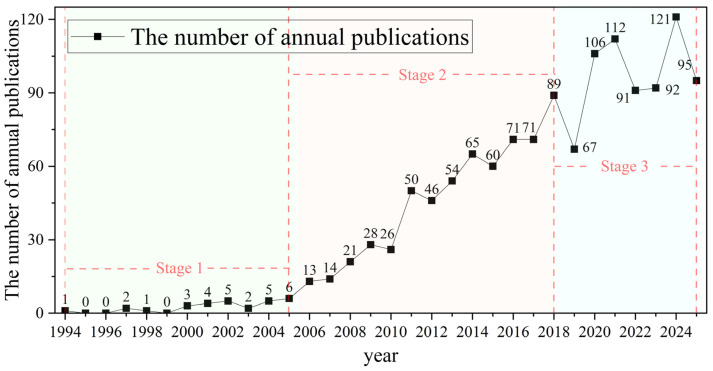
Annual publication statistics of geometric morphometrics in entomological research.

Systematic statistical analysis reveals developmental trends within a research field and provides insight into its dissemination and prevalence. In this study, we analyzed annual publication counts, publication output by country, and publication output by author. To enhance comparative interpretation, country-level and author-level publication data are further presented in the subsequent collaboration network analysis. Figure 2 presents the annual publication output of geometric morphometrics in entomological research. The field emerged around 1994. Over the subsequent 31 years, the annual number of publications has shown a fluctuating upward trend, reflecting changes in academic interest and research focus across different stages. From 1994 to 2005, the field was in its initial stage, with only 0–6 publications per year. During this period, geometric morphometrics was primarily applied to landmarking and the recording of biological images for early insect morphological classification [36]. Between 2006 and 2018, the field entered a rapid development phase, with annual publications increasing to 89. At this stage, a more systematic theoretical framework was established, with research largely focusing on variations in insect wing morphology. Additionally, several studies integrated geometric morphometrics with population genetics and phylogenetic approaches to investigate species evolution and genetic variation [37,38,39]. From 2019 to 2025, the field experienced fluctuating growth, with annual output varying but generally increasing. This stage witnessed notable advances in data acquisition, analytical methodologies, and interdisciplinary integration. In addition to traditional approaches such as one-way analysis of variance (ANOVA), principal component analysis (PCA), canonical variate analysis (CVA), thin-plate spline (TPS) analysis, multivariate analysis of variance (MANOVA), and cluster analysis, artificial intelligence (AI) and machine learning techniques have increasingly been incorporated into geometric morphometric research. For example, studies employing support vector machines (SVMs), random forest (RF), and artificial neural networks (ANNs) demonstrated that integrating machine learning algorithms with wing geometric morphometric data substantially improve the identification accuracy of cryptic and closely related species [40]. Meanwhile, deep learning models such as convolutional neural networks (CNNs) were widely applied to the automated classification of mosquito wing images, achieving high levels of species discrimination accuracy [41]. Furthermore, automated land-mark detection based on deep neural networks has become increasingly mature, enabling efficient and standardized landmark extraction in insect morphometrics while significantly reducing manual annotation error [42]. At the current stage, research increasingly integrates genetics, molecular phylogenetics, artificial intelligence, and species distribution models (SDMs), reflecting a clear trend toward interdisciplinary convergence and methodological intelligence [43,44,45].

### 3.2. Collaboration Network Analysis

#### 3.2.1. Country Collaboration Network Analysis

**Figure 3:**
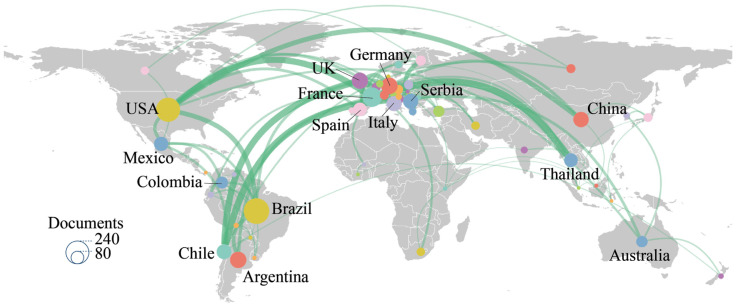
National Collaboration Network Map (The size of each node represents the publication output of the corresponding country. The thickness of the links indicates the strength of collaboration between countries, with thicker lines representing stronger collaborative relationships).

Table 1 and Figure 3 were generated using VOSviewer and Scimago Graphica, respectively, to present the publication output by country, co-occurrence network of publications, and collaboration network among countries in this field. Only countries with more than five publications were included. As shown in Table 1, the three leading countries in terms of publication output are Brazil (234 publications), the USA (209), and France (134). The USA, France, and the UK began research in this field relatively early, as reflected by their earlier average publication years. Brazil, the USA, and Germany have made sustained contributions over time, with publication years centered around 2018. In contrast, China and Mexico started later but have already achieved high publication output.

Figure 3 indicates that, although Brazil has the highest publication output, its international collaboration remains relatively limited. In contrast, the USA and France maintain collaborations with multiple countries worldwide. Chile primarily collaborates with France and the UK, whereas Thailand’s main collaborator is France. Overall, collaborations are concentrated among major countries such as the USA, France, the UK, Chile, and Thailand, as well as several smaller countries. For example, a study jointly conducted by researchers from the USA, France, Uruguay, and Ecuador, titled “Chagas vectors *Panstrongylus chinai* (Del Ponte, 1929) and *Panstrongylus howardi* (Neiva, 1911): chromatic forms or true species?”, employed geometric morphometrics and cytogenetic methods to demonstrate that the two disputed taxa are distinct species rather than conspecific forms [46]. Another study, “Geometric Morphometric Analysis of two genera confirm three new wasps from the mid-Cretaceous of Myanmar (Hymenoptera: Aulacidae)”, conducted by Gansu Agricultural University (China) and Wright State University (USA), applied geometric morphometric analysis to mid-Cretaceous amber fossils and confirmed three new species within two genera of the family Aulacidae [47].

**Table 1:** Statistics of publication output across countries.

Country	Freq	Burst	Degree	Centrality	Sigma	First Pub. Year	Avg. Pub. Year	Half-Life
Brazil	233	0	31	0.06	1	2005	2018	13.5
USA	209	0	55	0.32	1	1998	2017	19.5
France	134	7.03	46	0.24	4.46	2000	2015	15.5
UK	102	4.33	42	0.12	1.66	2001	2016	15.5
Germany	99	0	46	0.23	1	2006	2019	14.5
Argentina	95	0	21	0.02	1	2003	2018	15.5
China	86	0	29	0.09	1	2010	2019	10.5
Italy	83	8.68	32	0.09	2.09	2003	2015	11.5
Serbia	82	0	28	0.06	1	2008	2017	9.5
Chile	80	0	26	0.05	1	2003	2018	15.5
Mexico	74	7.35	21	0.04	1.34	2011	2020	10.5
Spain	67	0	38	0.06	1	2002	2018	17.5
Thailand	67	6.62	14	0.05	1.4	2007	2020	13.5
Colombia	51	0	15	0.01	1	2002	2016	13.5
Australia	47	3.6	28	0.1	1.39	2006	2017	10.5

Notes: Burst indicates a sudden increase in publication output during a specific period [48]. Degree is the number of collaborative links a country has with others [34]. Centrality refers to betweenness centrality, which measures the extent to which a country acts as a bridge in the collaboration network [49]. Sigma is a composite index integrating centrality and burst strength [34]. First pub. year refers to the year of the first publication. Avg. pub. year represents the average year of publication. Half-life is the time it takes for a country’s publication impact to decrease by half [34].

#### 3.2.2. Author Collaboration Network Analysis

**Figure 4:**
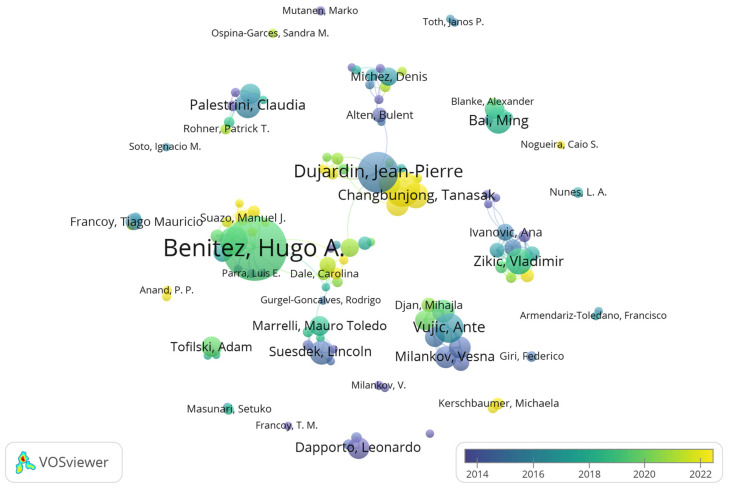
Author Collaboration Network Map.

Figure 4 shows the collaboration network of authors with more than five publications, generated using VOSviewer. Table 2 presents the top ten authors by publication output.

(1) The most prolific and extensively collaborative author in this field is Benitez, Hugo A., who applies geometric morphometrics to investigate morphological diversity across various insects taxa, exploring the mechanisms and evolutionary origins of morphological variation [50]. For example, he integrated geometric morphometrics, taxonomy, and molecular identification to investigate wing shape variation and environmental adaptation in *Phulia nymphula* (Blanchard, 1852) [51], and identified a new species, *Trichocera maculipennis*, highlighting the biosafety implications of its habitat and behavioral observations [52].

(2) Other highly productive and collaborative authors include Dujardin, Jean-Pierre; Chaiphongpachara, Tanawat; and Vujic, Ante, whose research primarily focuses on taxonomy and characterization using geometric morphometrics. For instance, Dujardin and colleagues employed geometric morphometrics to identify species and sex in larvae of *Chrysomya chani* Kurahashi, 2010 and *Chrysomya megacephala* (Fabricius, 1794), and compared head morphology of *Ctenocephalides felis* (Bouché, 1835) and *Ctenocephalides orientis* (Jordan, 1925), demonstrating that geometric morphometrics based on head shape curves provides a rapid and accurate tool for species and sex identification, useful for future epidemiological and demographic studies [53,54]. Additionally, a multinational study by Vujic et al. (2023) employed linear morphometrics and semi-landmark geometric morphometrics to analyze the shape of the R4+5 vein and wing morphology in the family Syrphidae Latreille, 1802 confirming the importance of R4+5 vein shape in distinguishing cryptic species and sexes within four *Merodon* species groups [55].

(3) In China, the field is primarily advanced by researchers such as Ming Bai and Dong Ren, who focus on improving insect taxonomy through digital geometric morphometric features. For example, a recent study combined quantitative morphometrics and statistical methods to analyze biodiversity within the phylogenetic framework of higher taxonomic units represented by the superfamily *Scarabaeoidea*, providing new insights into the selective pressures and mechanisms regulating species diversification and decline [56].

### 3.3. Keyword Co-Occurrence Network Analysis

**Figure 5:**
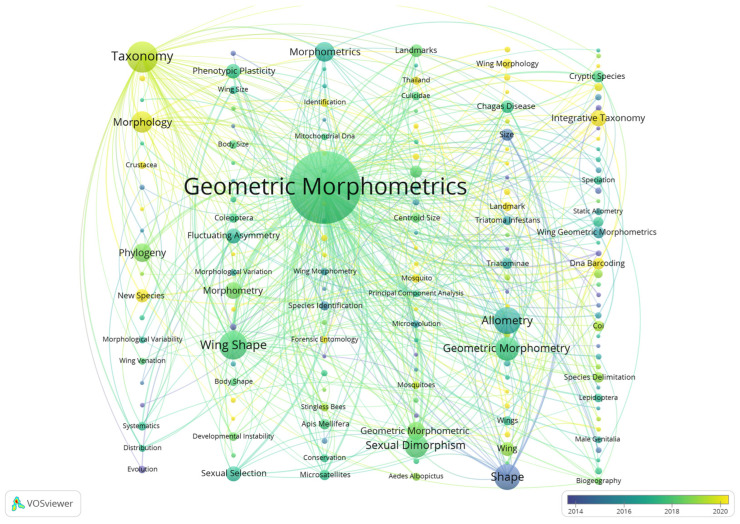
Keyword Co-occurrence Network Map (The size of each node represents the frequency of occurrence of the corresponding keyword; the links indicate that two keywords co-occurrence in the same article, with thicker lines denoting a stronger correlation between them; colors represent time periods).

Keywords accurately reflect the core content of articles, and analyzing high-frequency keywords within a field enables the identification of research hotspots and emerging trends [57,58,59]. The keyword co-occurrence network map and heatmap were generated (Figure 5), while Table 3 lists the top ten most frequent keywords. Only keywords with more than five occurrences were included in the analysis. Overall, 1321 relevant publications yielded 131 distinct keywords. Among the top ten, four are directly related to or synonymous with geometric morphometrics, together appearing 909 times. Excluding geometric morphometrics, the most frequent keywords were Taxonomy (123 occurrences), Wing Shape (116), Allometry (89), Shape (89), Sexual Dimorphism (79), and Phylogeny (54).

These results indicate that geometric morphometrics has become an essential tool in insect taxonomy, with much of the current research closely associated with taxonomic studies. For instance, some studies applied landmark data of head morphology in *Megalopta Smith* to distinguish sexes and population groups, while others used geometric morphometric datasets to classify 41 species within the family *Macrostylidae* [60,61]. Beyond classifications based on wing and head morphology, research on allometry, phylogeny, and sexual dimorphism has also emerged as major directions. For example, Casaubon et al. (2023) integrated molecular genetics with geometric morphometrics to reconstruct the phylogeny of the *Alpheus gracilipes* Stimpson, 1860 species complex, reporting new distribution sites and an expanded range for the first time [62]. Similarly, other scholars examined the wing, head, and body shape characteristics of the *Rhodnius prolixus* Stål, 1859 species group and engorged deutonymphs, assessing interspecific allometry, morphological differences, and their taxonomic significance [63,64].

### 3.4. Citation Network Analysis

**Figure 6:**
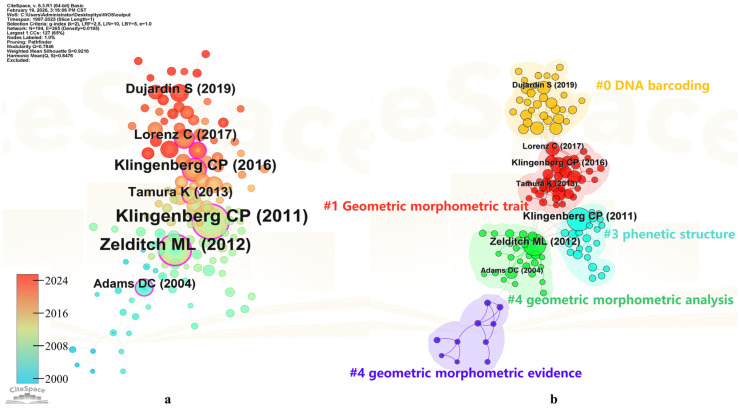
Co-citation network (**a**) and its cluster map (**b**). Nodes in (**a**) represent co-cited references, with node size proportional to co-citation frequency and colors indicating publication year. Purple outer rings denote high betweenness centrality in (**a**). Clusters in (**b**) represent major research themes and were labeled based on title terms extracted by CiteSpace.

A citation network reflects the frequency and centrality of co-cited publications within a field, serving as a measure of an article’s influence. By analyzing co-cited references, researchers can overcome the limitations of specific research directions and access more diverse data sources [58,65]. In this study, only references with more than one co-citation were included in the analysis. CiteSpace was used to construct the co-citation network and clustering map for this field (Figure 6a,b), and the top ten co-cited references are summarized in Table 4. As shown in Table 4 and Figure 6a, the three most frequently co-cited publications are Klingenberg CP (2011), Zelditch ML (2012), and Klingenberg CP (2016). Notably, two of the top three references were authored by the same researcher, Klingenberg CP, both exhibiting high citation frequency and degree centrality. The 2011 article by Klingenberg, titled “MorphoJ: an integrated software package for geometric morphometrics”, ranks first in co-citation frequency [66]. It introduces MorphoJ, a Java-based cross-platform software package for geometric morphometrics, providing analytical tools to integrate morphological, molecular, and ecological data, and summarizes the methods included in the software [66]. Another article by the same author, “Size, shape, and form: concepts of allometry in geometric morphometrics”, systematically compares two approaches to allometry in geometric morphometrics—the Gould–Mosimann school and the Huxley–Jolicoeur school. By analyzing the structure of morphospace and its effects on allometric representation and size correction, Klingenberg demonstrates that although the two approaches emphasize different aspects, they are logically compatible, offering flexible tools for studies of evolution and development [5]. Zelditch ML’s 2012 book “*Geometric Morphometrics for Biologists*” ranks second in co-citation frequency and first in degree centrality [67]. Written in an accessible style with abundant illustrations and case studies, the book updates both theoretical and practical aspects, incorporates the latest literature reviews and software guides, and provides biologists with the most current methods for geometric morphometric analysis [67].

Through analysis of the clustering map, the most influential core publications within each research theme cluster can be effectively identified and evaluated. As shown in Figure 6b, the co-cited references in this field can be broadly categorized into DNA barcoding, geometric morphometric traits, phenetic structure, geometric morphometric analysis, and geometric morphometric evidence. Among them, the most frequently co-cited article, Klingenberg CP (2011), is primarily cited in the context of phenetic structure, whereas Zelditch ML (2012) is predominantly cited for geometric morphometric analysis.

In summary, the co-citation network analysis provides a systematic overview of the multidimensional applications of geometric morphometrics in insect research and highlights the most influential publications in the field. These co-cited references clusters not only reflect the knowledge structure of insect geometric morphometrics but also, through their clustering characteristics and network relationships, reveal the flow and integration of knowledge across different research directions.

## 4. Discussion

### 4.1. Knowledge Framework

**Figure 7:**
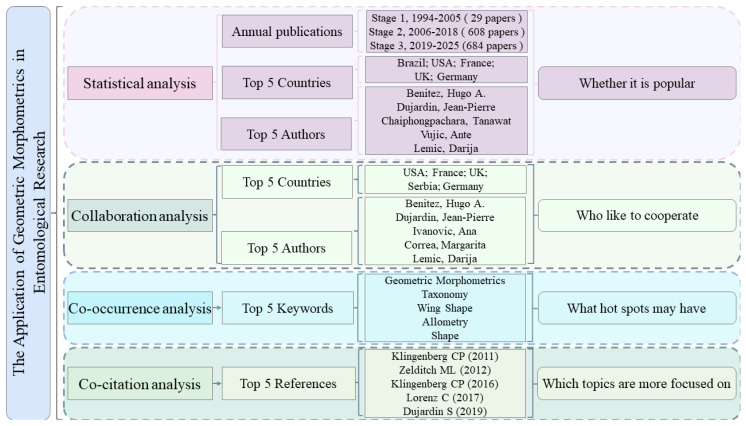
Knowledge Framework of Scientometrics.

Based on the above scientometric analysis, the development of geometric morphometrics in entomology reflects not merely a quantitative increase in publications, but also a structural transformation in methodological paradigms and research objectives (Figure 7).

The dominance of taxonomy based on wing shape is not coincidental. Insects possess rigid exoskeletons with conserved yet quantifiable morphological traits, particularly wings, which provide homologous landmarks suitable for Procrustes-based shape analyses. This anatomical suitability has positioned geometric morphometrics as an efficient, cost-effective alternative or complement to molecular identification. Therefore, the centrality of taxonomy in the keyword network reflects a broader disciplinary demand for rapid, standardized, and replicable tools for taxonomy. The co-citation structure further indicates a phase of methodological consolidation. The prominence of Klingenberg (2011; 2016) and Zelditch (2012) suggests that the field relies heavily on a relatively unified theoretical foundation, particularly regarding Procrustes superimposition, allometric theory, and morphospace interpretation. Such high intellectual centralization implies methodological maturity, but it may also suggest limited theoretical diversification compared to genomics-driven research domains. The collaboration network reveals a geographically asymmetric knowledge production system. The USA exhibits high betweenness centrality, functioning as a bridge within the global collaboration network. In contrast, Brazil, despite its high publication output, demonstrates relatively lower international connectivity. This pattern suggests that intellectual centrality is not determined solely by productivity, but also by structural positioning within collaborative networks.

Overall, the knowledge framework demonstrates that geometric morphometrics in entomology has evolved from a tool-oriented methodological innovation into a central analytical component of integrative taxonomy and evolutionary morphology. The field is gradually transitioning from isolated morphometric analyses toward cross-disciplinary integration involving molecular data, ecological contexts, and evolutionary theory.

### 4.2. Research Hotspots and Future Perspectives

**Figure 8:**
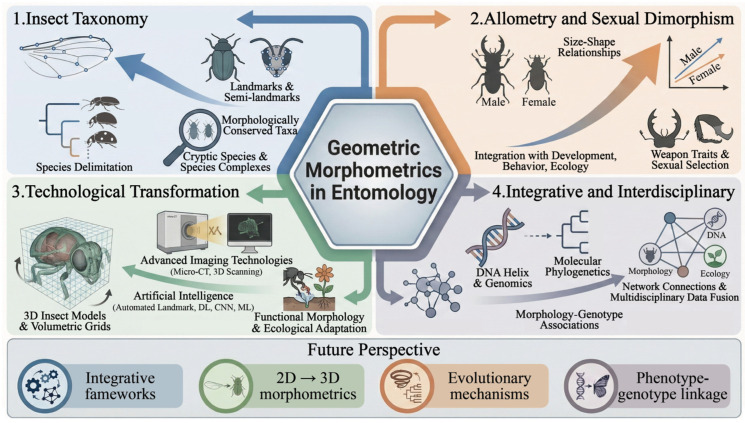
Research hotspots and future perspectives of geometric morphometrics.

Based on a systematic analysis of literature data from the past 31 years and the constructed knowledge framework, this study summarizes the current research hotspots and future perspectives of geometric morphometrics (Figure 8), aiming to provide conceptual insights and potential research directions for related research.

Geometric morphometrics has become a core analytical tool in insect taxonomy. This is reflected not only in the high-precision quantification of morphological traits, but also in its role in driving taxonomy from traditional descriptive identification toward quantitative species delimitation. Research on Diptera, Coleoptera, and Hymenoptera remains dominant [14], reflecting both the importance of these taxa in biodiversity research and their anatomical suitability for structural analysis. These insect wing structures are particularly well suited for Procrustes superimposition due to their homology and relatively stable morphological characteristics [68,69]. Empirical studies have demonstrated that geometric morphometrics can effectively distinguish conservative taxa and cryptic species complexes that are difficult to resolve using traditional morphometrics, while maintaining long-term stability in species identification models [70,71]. The widespread occurrence of cryptic diversity further strengthens the demand for high-resolution quantitative morphological tools. Future research will extend from single-structure analyses to multi-trait integrative frameworks that jointly incorporate wings, heads, genitalia, and overall body morphometrics, thereby enhancing the robustness and explanatory power of species delimitation.

Geometric morphometrics is increasingly applied to elucidate the evolutionary mechanisms underlying morphological variation, particularly in studies of allometry and sexual dimorphism. Compared with traditional morphometrics, it separates size and shape within multidimensional morphospace, allowing for more precise evaluation of the contribution of allometry to phenotypic differentiation. This methodological advantage makes it a powerful framework for testing hypotheses related to sexual selection, developmental constraints, and resource allocation strategies. Empirical evidence suggests that sexual dimorphism often exhibits continuous rather than discrete variation patterns [72,73]. Geometric morphometrics captures such gradual variation through morphospace structure, providing quantitative support for understanding weapon evolution, functional divergence, and ecological adaptation driven by sexual selection [72,74]. Furthermore, analyzing the systematic relationship between shape variation and body size facilitates differentiation between the effects of selective pressures and developmental constraints in morphological evolution [75]. Importantly, this approach contributes to a paradigm shift from merely describing differences to explaining underlying mechanisms. Future research should integrate developmental, behavioral, and ecological datasets to provide multidimensional evidence for clarifying mechanisms of sexual selection, adaptive evolution, and biodiversity conservation.

With the rapid advancement of imaging technology and computational capacity, geometric morphometrics is undergoing a methodological transformation from two-dimensional (2D) landmark analysis toward three-dimensional (3D) reconstruction, high-throughput data processing, and intelligent identification systems. Compared with 2D data, 3D data provide higher dimensional resolution and richer morphological information, overcoming the limitations of 2D analyses in capturing morphological features and resolving spatial structures [76,77]. In recent years, artificial intelligence (AI) and machine learning (ML) techniques have rapidly advanced. Automated landmark detection, deep learning (DL)-assisted morphological classification, and convolutional neural network (CNN)-based image feature extraction have substantially improved data-processing efficiency and classification accuracy [40,41,42]. These intelligent analytical workflows enhance repeatability and scalability in species identification and morphological comparison, while supporting large-scale biodiversity monitoring and digital specimen repository construction. In the future, with the establishment of multimodal data-integration platforms and cross-scale analytical frameworks, geometric morphometrics will likely play an increasingly prominent role in macroevolutionary research, biodiversity assessment, and ecological risk prediction.

The integration of geometric morphometrics with molecular biology, ecology, taxonomy, and phylogenetics is expected to become a key focus of future research. In classical phylogenetic studies, molecular data typically dominate due to their statistical robustness, whereas morphological data are often treated as secondary because of challenges in generating statistically compatible metrics [78]. By constructing repeatable, statistically testable morphospaces, geometric morphometrics enables phenotypic information to be incorporated as continuous variables into phylogenetic analyses, thereby enhancing the explanatory power of morphometrics in evolutionary interpretation [79]. At macroevolutionary scales, combining morphospace structure with phylogenetic frameworks facilitates the detection of convergent evolution, morphological conservatism, and differences in evolutionary rates among lineages [80,81]. This approach also allows for the visualization of phenotypic trajectories in adaptive radiations among lineages. This demonstrates that integrating geometric morphometrics with genomic data can more deeply reveal the genetic basis of morphological variation and its evolutionary mechanisms, while interdisciplinary approaches provide powerful tools to elucidate the genetic regulatory networks and evolutionary drivers underlying complex biological traits. In the future, with the deep integration of phylogenetics and geometric morphometrics, its theoretical importance will be further strengthened in identifying macroevolutionary patterns, investigating adaptive radiation, and interpreting biodiversity patterns.

Overall, the continued development of geometric morphometrics is reshaping entomological research at multiple levels, from species delimitation and evolutionary mechanism inference to methodological innovation and phylogenetic integration. Beyond serving as a precise tool for morphological quantification, it is increasingly embedded within data-driven and integrative analytical frameworks that bridge phenotype and genotype across scales. As 3D approaches, artificial intelligence, and comparative phylogenetic methods continue to converge, geometric morphometrics is poised to play a more central role in elucidating morphological evolution, adaptive diversification, and biodiversity patterns. Collectively, these developments signal a transition in entomology toward a more quantitative, integrative, and multidisciplinary paradigm.

### 4.3. Methodological Reflection and Limitations

This study represents a secondary research synthesis that outlines the development and structure of geometric morphometrics in entomology. As a meta-scientific review, it does not aim to test specific scientific hypotheses in entomology. Instead, it provides a structured and comprehensive overview of knowledge accumulation in the discipline by systematically analyzing publication patterns, collaborative networks, and thematic evolution. Such synthetic studies help clarify the intellectual landscape of the field, identify existing research gaps, and provide guidance for future primary research that is more targeted and impactful.

This scientometric study has some inherent limitations. The analysis is based on publications indexed in the Web of Science, which may lead to slight data-base coverage bias. In addition, only English-language publications were included, which may not fully represent global research output in this field.

## 5. Conclusions

By conducting a scientometric analysis of studies on geometric morphometrics in entomology, this study summarizes the developmental trends, research hotspots, and knowledge structure of the field over the past three decades, highlighting the important role of geometric morphometrics in modern entomological research. The results indicate that this approach has evolved from an early focus on basic taxonomic studies to become a key tool linking morphology, phylogeny, biodiversity, and evolutionary research, demonstrating particular advantages in addressing taxonomically challenging groups such as morphologically conserved taxa, cryptic species, and species complexes.

As research attention has expanded toward allometry, sexual dimorphism, and mechanisms of morphological evolution, geometric morphometrics has provided new quantitative perspectives for interpreting insect morphological diversification. Advances in three-dimensional geometric morphometrics, together with the increasing incorporation of artificial intelligence and machine learning techniques, have substantially enhanced the analysis efficiency, accuracy, and automation of complex morphological data, opening broader avenues for investigating insect ecological adaptation and evolutionary processes. Furthermore, the growing integration of geometric morphometrics with molecular phylogenetics, genomics, ecology, and intelligent analytical approaches facilitates a more comprehensive understanding of morphological variation from both phenotypic and genetic perspectives, promoting a shift in entomological research from descriptive morphology toward multi-scale, integrative, and data-driven research frameworks. This review can also provide important guidance for researchers in related fields in terms of method selection, study design, and interdisciplinary integration, thereby promoting further advances in insect biodiversity research and conservation practice.

## Figures and Tables

**Table 2:** Statistics of authors’ publication output and citation counts.

No.	Author	Documents	Citations	Avg. Pub. Year	Avg. Citations
1	Benitez, Hugo A.	60	612	2019	10.2
2	Dujardin, Jean-Pierre	34	879	2015	25.8529
3	Chaiphongpachara, Tanawat	29	180	2023	6.2069
4	Vujic, Ante	23	510	2016	22.1739
5	Lemic, Darija	22	311	2019	14.1364
6	Bai, Ming	20	259	2018	12.95
7	Zikic, Vladimir	20	190	2019	9.5
8	Changbunjong, Tanasak	19	141	2023	7.4211
9	Palestrini, Claudia	19	184	2016	9.6842
10	Suesdek, Lincoln	18	434	2015	24.1111

**Table 3:** Keyword Co-occurrence Statistics.

No.	Keyword	Freq	Avg. Pub. Year
1	Geometric Morphometrics	698	2017
2	Taxonomy	123	2019
3	Wing Shape	116	2018
4	Allometry	89	2016
5	Shape	89	2014
6	Geometric Morphometry	83	2017
7	Sexual Dimorphism	79	2018
8	Morphology	66	2019
9	Morphometrics	62	2017
10	Phylogeny	54	2018

**Table 4:** Co-cited References Statistics.

No.	Label	Author	Freq	Degree	Centrality
1	Klingenberg CP (2011)	Klingenberg CP	111	17	0.22
2	Zelditch ML (2012)	Zelditch ML	97	20	0.4
3	Klingenberg CP (2016)	Klingenberg CP	51	8	0.15
4	Lorenz C (2017)	Lorenz C	35	9	0.14
5	Dujardin S (2019)	Dujardin S	31	7	0.07
6	Tamura K (2013)	Tamura K	31	6	0.04
7	Adams DC (2013)	Adams DC	29	6	0.1
8	Klingenberg CP (2015)	Klingenberg CP	29	6	0.04
9	Baken EK (2021)	Baken EK	29	4	0.01
10	Adams DC (2013)	Adams DC	27	10	0.13

## Data Availability

The original contributions presented in the study are included in the article. Further inquiries can be directed to the corresponding author.
